# Global trends in research related to the links between microbiota and antibiotics: a visualization study

**DOI:** 10.1038/s41598-023-34187-8

**Published:** 2023-04-27

**Authors:** Sa’ed H. Zyoud, Muna Shakhshir, Amani S. Abushanab, Amer Koni, Adham Abu Taha, Faris Abushamma, Ali Sabateen, Samah W. Al-Jabi

**Affiliations:** 1 Poison Control and Drug Information Center (PCDIC), College of Medicine and Health Sciences, An-Najah National University, Nablus, 44839 Palestine; 2 Department of Clinical and Community Pharmacy, College of Medicine and Health Sciences, An-Najah National University, Nablus, 44839 Palestine; 3 Clinical Research Centre, An-Najah National University Hospital, Nablus, 44839 Palestine; 4 Department of Nutrition, An-Najah National University Hospital, Nablus, 44839 Palestine; 5 Division of Clinical Pharmacy, Hematology and Oncology Pharmacy Department, An-Najah National University Hospital, Nablus, 44839 Palestine; 6 Department of Biomedical Sciences, Faculty of Medicine and Health Sciences, An-Najah National University, Nablus, 44839 Palestine; 7 Department of Pathology, An-Najah National University Hospital, Nablus, 44839 Palestine; 8 Department of Medicine, College of Medicine and Health Sciences, An-Najah National University, Nablus, 44839 Palestine; 9 Department of Urology, An-Najah National University Hospital, Nablus, 44839 Palestine; 10 Infection Control Department, An-Najah National University Hospital, Nablus, 44839 Palestine

**Keywords:** Microbiology, Antimicrobials, Medical research

## Abstract

The scientific community widely acknowledges that the gut microbiota plays a critical role in maintaining host health and can be altered by a range of factors, such as antibiotic use, diet, stress, and infections. Therefore, this study utilized bibliometric analysis to thoroughly investigate research trends in the microbiota and antibiotics. Scopus was used to extract papers linked to microbiota and antibiotics published between 2002 and 2021, and both Microsoft Excel and VOSviewer were used to conduct the analysis of the data. A total of 2,816 publications discussed the connection between the microbiota and antibiotics. Growth occurred in two stages: the first (2002–2015) was characterized by fairly slow publication production, while the second (2016–2021) saw a rapid increase in publishing progress. The United States has the most publications, 654, representing 23.22% of the total. China came second with 372 publications (13.21%), followed by the United Kingdom with 161 publications (5.72%) and India with 157 publications (5.58%). In addition, publications on ‘altered intestinal microbiota composition with antibiotic treatment’ were introduced after 2017, while ‘gut microbiota and antimicrobial resistance’ and ‘probiotics as an alternative antimicrobial therapy’ were introduced before 2017. Based on these results, this study provides an in-depth look at key moments in the history of microbiota and antibiotic research, as well as possible directions for future research in different areas of microbiota and antibiotic research. Therefore, it is suggested that more attention should be given to the latest promising hotspots, such as how antibiotic treatment changes the composition of the gut microbiota.

## Introduction

Many microbes, including viruses, bacteria, fungi, protozoa, and archaea, colonize the human gastrointestinal tract (GI), termed the gut microbiota^1^. Research in recent decades has focused on and linked the gastrointestinal microbiome to many diseases and human health^2^.

The gut microbiota protects the GI tract against infection using different mechanisms that contribute to resistance against colonization by exogenous microorganisms and protection against fatal pathogens^3^. Colonization resistance may occur directly through competitive bacterial interaction or host defense against pathogens, which is triggered indirectly by bacteria. Many studies have shown not only the association but also the causality of many diseases with gut microbiomes. Furthermore, studies have revealed that intrinsic and extrinsic factors, mainly drugs, can affect gut microbiome function and/or composition, ultimately affecting human health^2^.

The importance of the gut microbiome, as well as its connection to health, the immune system, and the use of antibiotics^4–6^, has been brought to light by research conducted in recent years. Because research output plays an important function in science development by providing a key association between the production of knowledge and its application, the volume of research that encompasses almost all of the world’s regions that are interested in the production of health sciences is growing. This is one of the reasons why there is a growing interest in the production of health sciences. Despite the fact that only a few bibliometric studies have been conducted in the field of gut microbiota^7–11^, none of them have investigated the links between the microbiota and antibiotics. To fill this gap, the authors of this study used bibliometric analysis to thoroughly investigate the key research trends related to microbiota and antibiotics. Bibliometric analysis refers to the quantitative and qualitative examination of the literature in a specific field utilizing statistical and mathematical methods. Over the past decade, numerous studies have been conducted to explore bibliometrics in various scientific disciplines^12–14^. Unlike systematic reviews, which aim to answer a particular research question based on a limited number of publications, bibliometric analysis aims to provide a comprehensive overview of the literature in a particular field^15^. Similarly, it differs from scoping reviews, which focus on identifying the nature and scope of research evidence^16^. Despite these differences, bibliometric analysis remains a valuable tool for obtaining a snapshot of both national and international contributions to the literature in a particular field, as well as identifying research gaps that future studies may address^13,14^. By investigating the global links between the research dynamics and hotspots of the microbiota and antibiotics, this paper aims to gain insight into the research trends of the microbiota and antibiotics to provide references for future research. Furthermore, by gaining insights into the research trends and hotspots in the field, policymakers and funding agencies can make informed decisions about where to allocate research resources and prioritize future research efforts.

## Methods

### Source of data

In bibliometric analysis, documents are retrieved from a single database and then analyzed both quantitatively and qualitatively^14,17^. Typically, either SciVerse Scopus or Web of Knowledge is used as the single database, and no gray literature is included in the analysis. For this particular study, publications relevant to microbiota and antibiotics from 2002 to 2021 were retrieved using SciVerse Scopus, which was chosen due to its many advantages over other databases, such as Web of Science, Medline, and Google Scholar^18,19^. One of the key features of Scopus is its ability to provide bibliometric indicators in a direct and simple way. Additionally, Scopus includes 100% of Medline's publications, so by using Scopus, publications in Medline are automatically included as well. Many academics have extensively used the Scopus database, one of the most complete, systematic, and reliable databases, for bibliometric analysis and visualization of the scientific literature. Because the Scopus database is always accessible, we decided to perform the literature retrieval from it on a single day, December 1, 2022, to eliminate any potential for bias that daily additions to the database could have caused.

The terms related to the microbiota and antibiotics were identified using PubMed Medical Subject Headings (MeSH) and relevant publications on the topics (microbiota^20–22^ and antibiotics^23–26^). These terms were then included in the Scopus Engine. Each of the chosen “keywords” was used as an entry for the “Article Title”. The keywords used in the study, ‘antibiotics’ or ‘antimicrobials’, ‘antibiotics’ or ‘antimicrobials’, were those related to antibiotics per se rather than other related terminology, such as specific name or class of antibiotics.

### Validation of the search strategy

After refining the search query, it was ensured that there would be no false positive results by determining whether the most frequently cited publications (i.e., the top 100) were relevant to the topic being looked up. The author contacted two bibliometrics professionals and requested that they verify false-positive results by reviewing the titles and abstracts of the most-cited documents that were supplied to them. When experts established that there were no false positive results, the search query was deemed to have reached its conclusion. The correlation test was implemented between the information retrieved by the search query and the real findings for the top ten active researchers to validate that there are no false-negative results. The fact that the correlation test yielded a strong correlation (r = 0.952) and a significant result (p < 0.01) provides evidence that the search query was accurate. This particular validation method has been used in bibliometric investigations carried out in the past and published in the literature^27,28^.

### Data export

The data retrieved were transferred to Microsoft Excel for analysis and tabulation. Export data included types of retrieved documents, the annual growth of publications, prolific countries, prolific institutions, prolific funding agencies, the most cited publications, and journals involved in publishing the retrieved documents. The retrieved data were also exported to VOSviewer v.1.6.18 (https://www.vosviewer.com/), which is a free online program that can be used for mapping purposes^29,30^. The VOSviewer application was used to create network visualization maps that presented international research collaboration and research hotspots.

### Ethics approval and consent to participate

There was no need for ethical approval because the data for the bibliometric research were extracted directly from the database without further human intervention.

## Results

### Description of the retrieved publications

Between 2002 and 2021, a total of 2816 publications discussed the connection between the microbiota and antibiotics. Of these, there were 2,225 (79.01%) original articles, 292 reviews, and 299 (10.62) other documents, including letters, conference papers, and editorials.

### Growth and productivity trends

**Figure 1:**
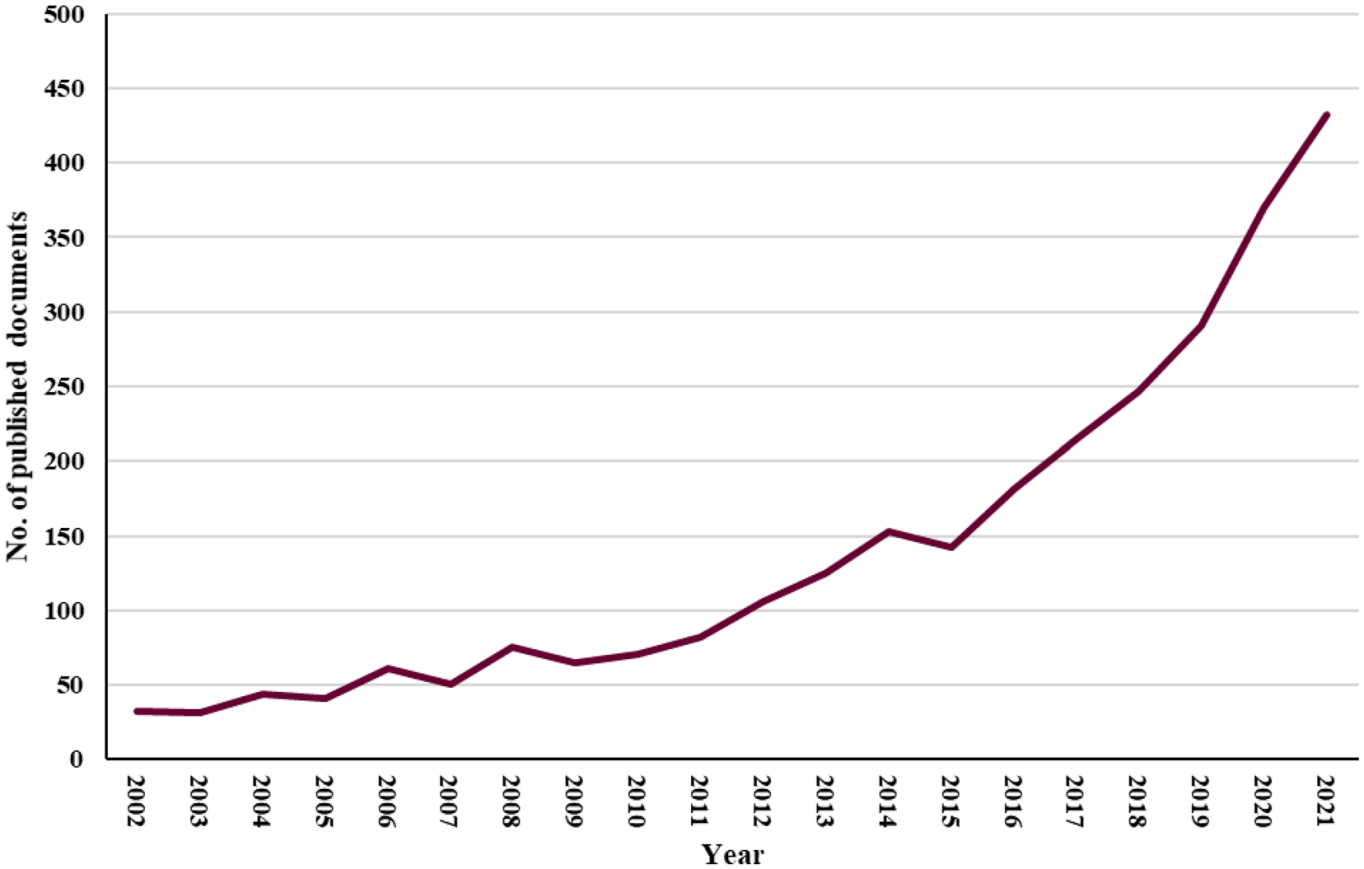
Trends in publications for research on the microbiota and antibiotics between 2002 and 2021.

Figure 1 shows the annual growth in the number of publications on microbiota and antibiotics in the past 20 years, from 32 in 2002 to 432 in 2021. Growth occurred in two stages: the first (2002–2015) was characterized by fairly slow publication production, while the second (2016–2021) saw a rapid increase in publishing progress.

### Performance of countries/regions

**Figure 2:**
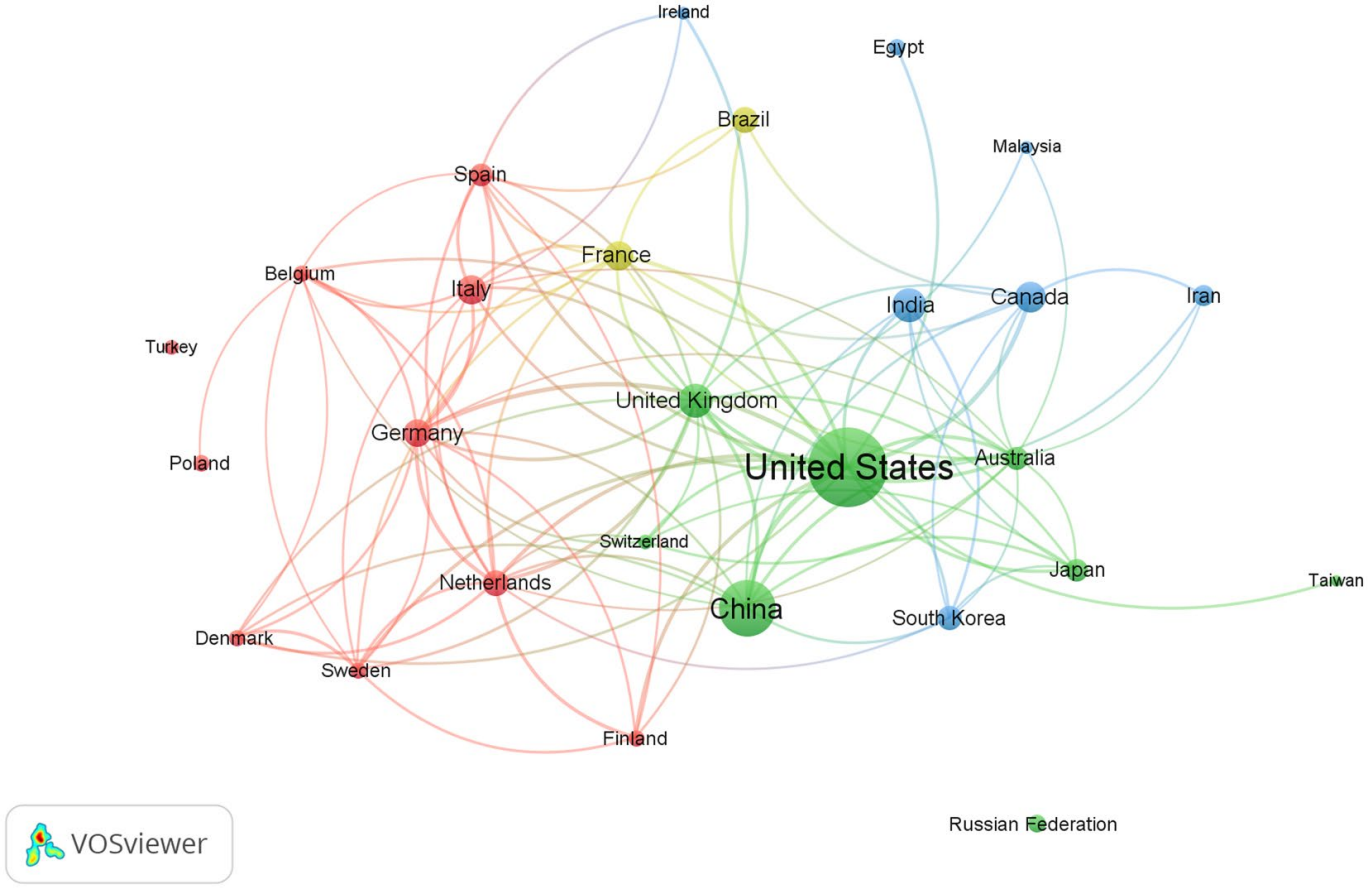
International (cross-country) research collaboration between countries with a minimum contribution of 30 articles. There were 27 countries on the map.

Table 1 lists the top ten countries in terms of research activity in this field. According to VOSviewer, a total of 2.816 publications were published in 110 countries, with 1802 of those publications published in the ten countries with the most research activity. The top 10 countries accounted for 63.99% of the total publications, with the United States having the most, with 654, which accounted for 23.22% of the total publications. China came second with 372 publications (13.21%), followed by the United Kingdom with 161 publications (5.72%) and India with 157 publications (5.58%). Figure 2 shows the international research collaboration between countries with a minimum contribution of 30 articles. There were 27 countries on the map. The countries in the center, including the United States, the United Kingdom, Italy, and Germany, had the most documents with international collaboration and the largest node sizes on the map.

**Table 1:** Top ten active countries in research related to the links between the microbiota and antibiotics from 2002 to 2021.

Ranking	Country	No. of documents	%
1st	United States	654	23.22
2nd	China	372	13.21
3rd	United Kingdom	161	5.72
4th	India	157	5.58
5th	Canada	144	5.11
6th	Italy	126	4.47
7th	France	124	4.40
7th	Germany	124	4.40
9th	Netherlands	103	3.66
10th	Brazil	100	3.55

### Contribution of institutions

The top 10 institutions in terms of the amount of research they have conducted in this area are listed in Table 2. According to Table 2, the *Ministry of Education of China* and the *Chinese Academy of Sciences* published the highest number of articles, with 35 publications each. Next came *Wageningen University & Research* with 33 publications and *INSERM* with 30 publications.

**Table 2:** Top ten active institutions in research related to the links between the microbiota and antibiotics from 2002 to 2021.

Ranking	Institute	Country	No. of documents	%
1st	*Ministry of Education China*	China	35	1.24
1st	*Chinese Academy of Sciences*	China	35	1.24
3rd	*Wageningen University & Research*	Netherlands	33	1.17
4th	*INSERM*	France	30	1.07
5th	*Københavns Universitet*	Denmark	29	1.03
6th	*China Agricultural University*	China	27	0.96
6th	*University of Alberta*	Canada	27	0.96
8th	*Zhejiang University*	China	25	0.89
9th	*Ministry of Agriculture of the People's Republic of China*	China	24	0.85
10th	*Washington University School of Medicine in St. Louis*	USA	23	0.82

### Contribution of funding agencies

Table 3 lists the top ten funding agencies in terms of the total amount of research they have supported in this field. Most of the funding agencies came from the United States, as shown in Table 3. *The National Natural Science Foundation of China* supported the highest number of articles, with 178 publications, followed by the *National Institutes of Health* with 162 publications and the *National Institute of Allergy and Infectious Diseases* with 83 publications.

**Table 3:** Top ten active funding agencies for research related to the links between the microbiota and antibiotics from 2002 to 2021.

Ranking	Institute	Country	No. of documents	%
1st	*National Natural Science Foundation of China*	China	178	6.32
2nd	*National Institutes of Health*	USA	162	5.75
3rd	*National Institute of Allergy and Infectious Diseases*	USA	83	2.95
4th	*National Institute of Diabetes and Digestive and Kidney Diseases*	USA	69	2.45
5th	*National Institute of General Medical Sciences*	USA	49	1.74
6th	*National Key Research and Development Program of China*	China	42	1.49
7th	*National Science Foundation*	USA	39	1.38
8th	*National Cancer Institute*	USA	37	1.31
9th	*Seventh Framework Programme*	European Union	32	1.14
10th	*Conselho Nacional de Desenvolvimento Científico e Tecnológico*	Brazil	30	1.07
10th	*National Center for Advancing Translational Sciences*	USA	30	1.07

### Contribution of journals

Table 4 contains a list of the ten journals that are considered the most productive in general. Most papers were published in this field by *Plos One* (69 documents, 2.45%), followed by *Frontiers in Microbiology* (55 documents, 1.95%), *Scientific Reports* (49 documents, 1.74%), and *Microorganisms* (29 documents, 1.03%). With an IF of 16.837, *Microbiome* was the most influential journal of the top 10 prolific journals.

**Table 4:** Top ten active journals for research related to the links between the microbiota and antibiotics from 2002 to 2021.

Ranking	Journal/source title	No. of documents	%	IF^a^
1st	*Plos One*	69	2.45	3.752
2nd	*Frontiers in Microbiology*	55	1.95	6.064
3rd	*Scientific Reports*	49	1.74	4.996
4th	*Microorganisms*	29	1.03	4.926
5th	*Antibiotics*	23	0.82	5.226
5th	*Microbiome*	23	0.82	16.837
7th	*Animals*	21	0.75	3.231
8th	*Antimicrobial Agents and Chemotherapy*	20	0.71	5.938
9th	*Gut Microbes*	19	0.67	9.434
10th	*Anaerobe*	18	0.64	2.837

^a^Journal Citation Reports (Clarivate, 2022).

### Analysis of highly cited publications

The 10 most cited publications on the microbiota and antibiotics are shown in Table 5. The number of citations in the top ten ranged from 1695 to 686^31–40^. The work that received the most citations in this area was written by Dethlefsen et al.^35^ and published in PLoS Biology. The works by Dethlefsen and Relman^37^ and Cho et al.^34^ received the second and third most citations, respectively.

**Table 5:** Top ten articles highly cited for research related to the links between the microbiota and antibiotics from 2002 to 2021.

Ranking	Authors	Title	Year	Source title	Cited by
1st	Dethlefsen et al.^35^	“The pervasive effects of an antibiotic on the human gut microbiota, as revealed by deep 16 s rRNA sequencing”	2008	*PLoS Biology*	1695
2nd	Dethlefsen and Relman^37^	“Incomplete recovery and individualized responses of the human distal gut microbiota to repeated antibiotic perturbation”	2011	*Proceedings of the National Academy of Sciences of the United States of America*	1480
3rd	Cho et al.^34^	“Antibiotics in early life alter the murine colonic microbiome and adiposity”	2012	*Nature*	1105
4th	Vaishnava et al.^40^	“The antibacterial lectin RegIIIγ promotes the spatial segregation of microbiota and host in the intestine”	2011	*Science*	937
5th	Sartor^31^	“Therapeutic manipulation of the enteric microflora in inflammatory bowel diseases: Antibiotics, probiotics, and prebiotics”	2004	*Gastroenterology*	869
6th	Jakobsson et al.^36^	“Short-term antibiotic treatment has differing long- term impacts on the human throat and gut microbiome”	2010	*PLoS ONE*	750
7th	Jernberg et al.^33^	“Long-term impacts of antibiotic exposure on the human intestinal microbiota”	2010	*Microbiology*	699
8th	Bokulich et al.^39^	“Antibiotics, birth mode, and diet shape microbiome maturation during early life”	2016	*Science Translational Medicine*	697
9th	Jernberg et al.^32^	“Long-term ecological impacts of antibiotic administration on the human intestinal microbiota”	2007	*ISME Journal*	696
10th	Looft et al.^38^	“In-feed antibiotic effects on the swine intestinal microbiome”	2012	*Proceedings of the National Academy of Sciences of the United States of America*	686

### Research hotspots

**Figure 3:**
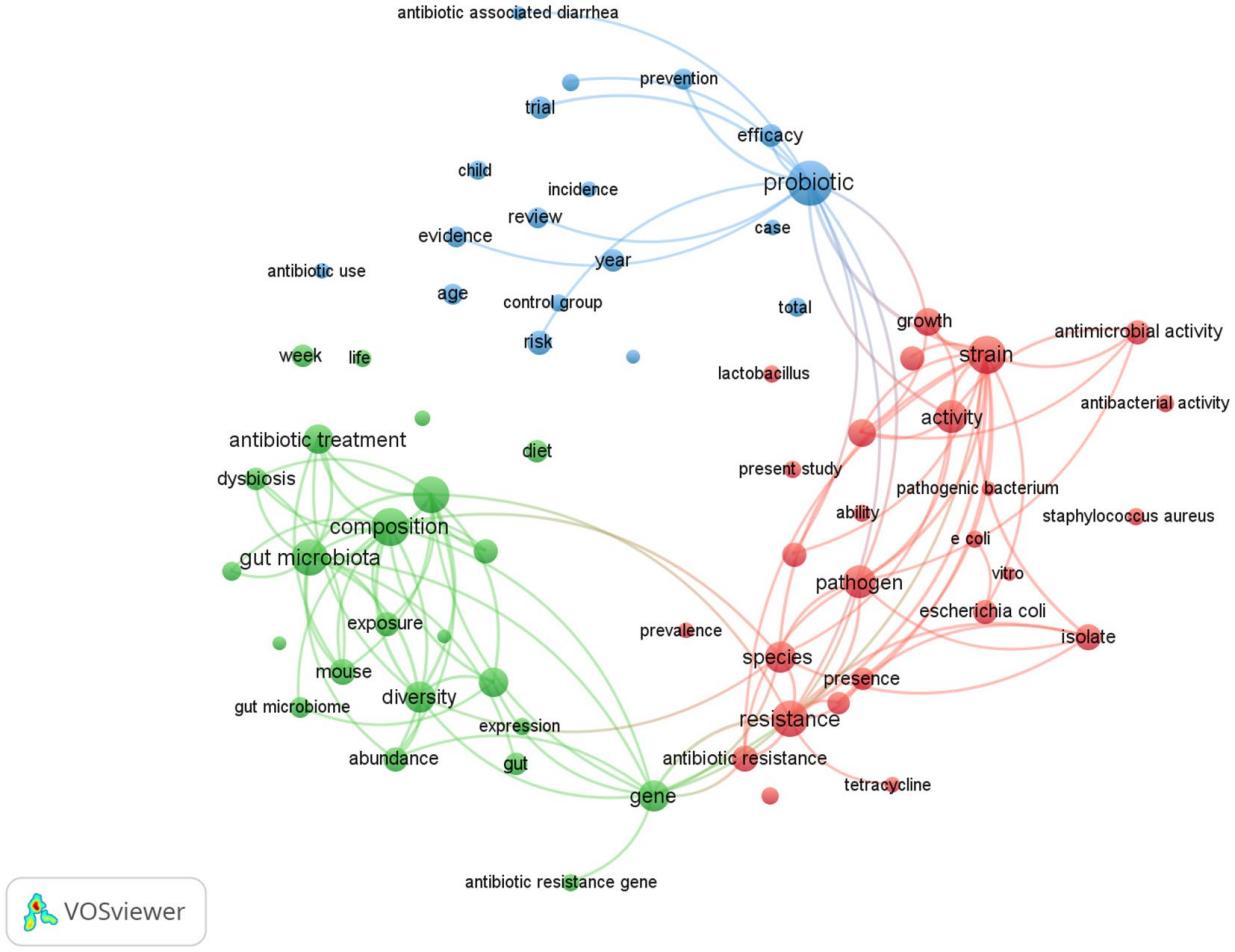
Research topics clustered by mapping the cooccurrences of terms for publications on microbiota and antibiotics. Of the 49,251 terms, 111 terms occurred at least 100 times.

**Figure 4:**
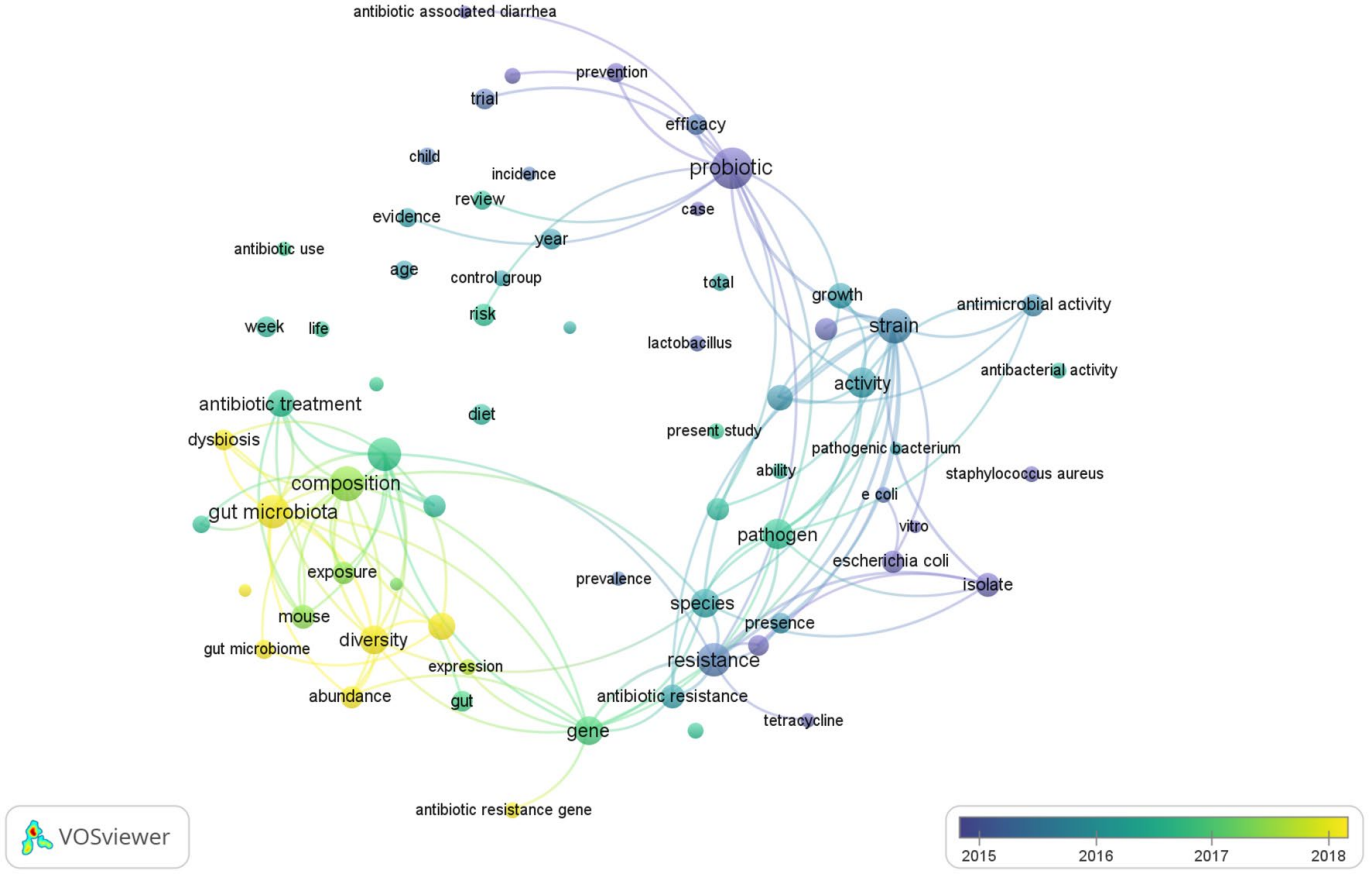
Overlay visualization map of the time sequence of frequently used terms in the microbiota and antibiotics (2002–2021). Yellow terms represent the most recent research.

By mapping the cooccurrences of terms in articles on microbiota and antibiotics, research areas were grouped into 3 clusters. There were 49,251 terms, and 111 of those terms appeared at least 100 times. The visual network map reveals that three clusters can be formed from all of these terms (Fig. 3): Cluster 1 (“altered gut microbiota composition with antibiotic treatment”, green nodes); Cluster 2 (“gut microbiota and antimicrobial resistance”, red nodes); and Cluster 3 (“probiotics as an alternative antimicrobial therapy”, blue nodes). Furthermore, the overlay visualization of the terms used to map the time sequence of these terms showed that the group related to altered gut microbiota composition with antibiotic treatment was introduced after 2017, while the other terms featured on the map were introduced before 2017 (Fig. 4).

### The most frequent antibiotic occurrences in the microbiota literature

Table 6 presents a list of antibiotics that are commonly mentioned in the microbiota literature, with “vancomycin” being the most frequently encountered (*n* = 314), followed by "ampicillin" (*n* = 258), "ciprofloxacin" (*n* = 244), and "metronidazole" (*n* = 233).

**Table 6:** List of the top ten frequent antibiotic occurrences in the microbiota literature from 2002 to 2021.

Ranking	Antibiotic	Frequency	%	Affecting microbial species, outcomes
1st	Vancomycin	314	11.15	Although vancomycin is commonly regarded as the most effective treatment for *C. difficile* infection (CDI), its use, as other antibiotics, has been linked to alterations in the gut microbiota that can lead to recurrent *C. difficile* infection (rCDI) or promotion of pathogenic strains of *E. coli*. Furthermore, Vancomycin treatment has been shown to result in depletion of several key intestinal microbiota, including *Bacteroidetes*, and is correlated with an increase in *Proteobacteria* species and a decrease in *Bacteroidetes*, *Fuminococcus* and *Faecalibacterium*^60,61^
2nd	Ampicillin	258	9.16	The use of ampicillin as a treatment led to a reduction in the population of bacterial genera containing beneficial strains, including *Coprococcus* and *Lactobacillus*, and an increase in the population of genera with potentially harmful strains, such as *Enterococcus*. Previous research has indicated that the decrease in the proportion of beneficial bacteria after taking antibiotics is related to a decrease in overall microbiota diversity and lower levels of beneficial bacteria^60^
3rd	Ciprofloxacin	244	8.66	Ciprofloxacin has a variable effect on anaerobic bacteria, such as *clostridia*, bifidobacteria, and *Bacteroides* spp.^62^. These bacteria are essential to maintain a healthy gut microbiome and perform critical functions such as breaking down complex carbohydrates and producing essential vitamins
4th	Metronidazole	233	8.27	The use of ampicillin as a treatment led to a reduction in the population of bacterial genera containing beneficial strains, including *Coprococcus* and *Lactobacillus*, and an increase in the population of genera with potentially harmful strains, such as *Enterococcus*. Previous research has indicated that the decrease in the proportion of beneficial bacteria after taking antibiotics is related to a decrease in overall microbiota diversity and lower levels of beneficial bacteria^60^
5th	Tetracycline	208	7.39	Tetracycline can reduce the abundance of beneficial bacteria in the gut microbiome, including *Lactobacillus* and *Bifidobacterium*. These bacteria are important to maintain a healthy digestive system, produce important vitamins and other compounds, and regulate the immune system
6th	Amoxicillin	190	6.75	The findings of the study indicate that there is a correlation between the use of antibiotics and changes in the composition of the gut microbiome. Specifically, the study confirmed that the abundance of *Bacteroidetes* increased during amoxicillin treatment, while the abundance of *Firmicutes* decreased. This change in the *Firmicutes*/*Bacteroidetes* ratio has been linked to various health outcomes such as increased BMI, obesity, and metabolic disease^63^
7th	Gentamicin	186	6.61	The abundance of *Bacteroidetes* and *Firmicutes*, two major phyla of bacteria in the intestine, was significantly reduced after gentamicin treatment. Furthermore, the study found that gentamicin treatment led to an increase in the abundance of *Proteobacteria*, a phylum of bacteria that is often associated with inflammation and disease^64,65^
8th	Clindamycin	157	5.58	Research has shown that clindamycin can lead to the overgrowth of certain types of bacteria, such as *Clostridium difficile*. This can cause an infection called *C. difficile* colitis, which can be serious and difficult to treat^66^
9th	Erythromycin	147	5.22	It has been shown that erythromycin can cause a decrease in the number of beneficial bacteria such as *Lactobacillus* and *Bifidobacterium*, which play an important role in maintaining gut health^67^. This can lead to the overgrowth of harmful bacteria, such as *Clostridium difficile*, which can cause diarrhea and other digestive problems^68^
10th	Neomycin	107	3.80	The use of ampicillin as a treatment led to a reduction in the population of bacterial genera containing beneficial strains, including *Coprococcus* and *Lactobacillus*, and an increase in the population of genera with potentially harmful strains, such as *Enterococcus*. Previous research has indicated that the decrease in the proportion of beneficial bacteria after taking antibiotics is related to a decrease in overall microbiota diversity and lower levels of beneficial bacteria^60^

## Discussion

To date, studies have revealed a link between the microbiota and several neurological and digestive diseases. The gut microbiota has become a focus of intense investigation in various diseases during the past two decades due to the growing interest in the function of intestinal microbe alterations in the pathogenesis of diseases. The status and tendencies of the gut microbiota in particular subjects are increasingly being investigated using bibliometric analysis^7,21,41–43^. However, no bibliometric research has been conducted on the links between antibiotic use and the microbiota. In the current study, we analyzed global trends and research horizons on the microbiota and antibiotics through bibliometric analysis to look back over the past 20 years. Researchers were shown to have a significant interest in microbiota and antibiotics, as evidenced by the increasing number of annual publications. The United States held the leadership position in microbiota and antibiotics with regard to publications and collaboration. However, this position cannot be separated from its substantial support from financial institutions.

The results of this study indicate that the United States, China, the United Kingdom, and India have made the most progress in microbiota-related antibiotic research in the past two decades, with the United States leading the way. This research is critical to combating antibiotic resistance, which is a global concern. It is essential that countries continue to invest in scientific research to develop new strategies for the use of antibiotics and combat this issue. According to the results, the United States has been at the forefront of antibiotic research trends^24–27,44^. This is likely due to several factors, including the availability of research funding, a strong infrastructure for scientific research, and a robust pharmaceutical industry. The United States is also home to several prestigious universities and research institutions that attract talented researchers and scientists from all over the world.

China ranked second in microbiota-related antibiotic research trends, indicating that the country has made significant strides in this field in recent years. China has a large population and has been hard hit by antibiotic-resistant infections^23,24,27,44^, making this research a priority. In addition, the country has invested heavily in scientific research in recent years and has a growing pharmaceutical industry.

The United Kingdom ranked third in the results, indicating that the country has also been active in microbiota-related research^22,45,46^. The UK has a long history of scientific research and innovation and is home to several renowned universities and research institutions.

India ranked fourth in the results, indicating that the country has progressed in microbiota-related antibiotic research. India is home to a large population with a high burden of infectious diseases^26^, making this research a priority. However, India faces several challenges in the field of scientific research, including limited funding, a lack of infrastructure, and brain drain, where talented scientists leave the country for better opportunities elsewhere.

The current study identified three research themes related to antibiotic use and microbiota. These research themes were closely related. The research theme that focused on the *altered composition of the gut microbiota with antibiotic treatment* was particularly interesting in the current study. Treatment with antibiotics has been shown to decrease the diversity of bacteria in the intestinal microbiome, causing metabolic changes that increase the potential subsequent susceptibility of the intestinal tract to colonization. This allows exogenous pathogens to invade the GI tract and cause infection, in addition to the development of antibiotic resistance^5,47,48^. Antibiotics may also disrupt the normal balance between the gut microbiota and various species. For example, *Clostridium difficile* may result from the antibiotic effect of antibiotics on decreasing species diversity^49^. Administration of a combination of gentamicin, meropenem, and vancomycin in adults resulted in a decrease in Bifidobacterium and butyrate-producing species and an increase in the prevalence of Enterobacteriaceae during the first treatment, which was then partially restored within the next month, while some bacterial species remained undetectable for longer periods in the gut^50^.

In the current study, ‘gut microbiota and antimicrobial resistance’ was among the most popular hot topics in research on the links between antibiotic use and the gut microbiota. Antibiotic resistance genes are present in the human gut microbiome. The number of resistance genes in the stomach increases rapidly during antibiotic therapy and gradually decreases once treatment ends^51^. Intestinal bacteria resistant to antibiotics can be passed from mother to child during birth and may remain for weeks. Twelve percent of the commensal E. coli bacteria in the Swedish study were positive for tetracycline resistance despite never being exposed to antibiotics^52^. As bacteria sensitive to the antibiotic are eliminated, resistant bacteria for the same antibiotic start to take their place by multiplying. After antibiotic treatment, the diversity of the bacterial species is reduced, while the total microbial load may increase. For example, evidence showed that patients treated with a week of b-lactams doubled the fecal microbial load in fecal samples^47^. Even if transient, colonization with multidrug-resistant bacteria can lead to the transfer of antibiotic resistance genes into the microbiota, which can eventually result in increased resistance to a variety of effective antibiotics and an increased risk of fatal infection^53^.

Another topic that has received much attention is ‘probiotics as an alternative antimicrobial therapy’. There is evidence that probiotics may help treat and prevent infectious diseases^54^. Although probiotic treatment also alters the luminal microbiota, this luminal alteration of the microbiota by probiotics could significantly influence systemic metabolism, including insulin resistance^55^. Multidrug-resistant bacteria, such as carbapenemase-producing enterobacteria, extended-spectrum beta-lactamase strains and vancomycin-resistant enterococci, are all serious pathogens that cause a high mortality rate and are considered an important public health problem^56^. Therefore, strategies to prevent the colonization of the luminal microbiota, especially in the colon, could be achieved using probiotics. However, little is known about their biological action despite substantial research on probiotics in recent years^57^.

The results of our research demonstrate that several subtopics closely related to study hotspots were highlighted in the most cited publications on the links between antibiotics and the microbiota^31–40^. Furthermore, these results demonstrate the increased emphasis and attention given to this area of research in recent years. For example, the most frequently cited article on the subject is published in *PLoS Biology* and has been cited 1695 times. This investigation used pyrosequencing technology to identify changes that occur in the microbiota population of three individuals before and after ciprofloxacin treatment^35^. The study clearly showed how ciprofloxacin impacted the diversity of the bacterial population in the intestine and its variability and balance. Furthermore, numerous taxa were unable to return to normal after six months of treatment^35^. The paper by Dethlefsen and Relman^37^, which was published in *Proceedings of the National Academy of Sciences of the United States of America*, was the second most cited article. This article was conducted to test the influence of the use of ciprofloxacin antibiotic on the communities of normal intestinal flora by studying approximately 1.7 million hypervariable 16S rRNA tags of 16S rRNA of the bacteria^37^. Ciprofloxacin greatly affected the bacterial population within 3–4 days of treatment. However, these populations began to recover after one week of stopping antibiotics, and although the recovery was imperfect, they did not return to their former status^37^.

The third most cited paper was published in *Nature*^34^. Again, the investigators adopted an animal model to examine the effect of administering a low-dose antibiotic to a young murine on the composition of the gut microbiome and its metabolic pathway. As a result, several changes were documented. An example of these changes is the alteration of essential genes responsible for converting carbohydrates to short-chain fatty acids^34^.

The paper by Vaishnava et al.^40^, published in *Science*, was the fourth most cited article. This study showed how antibacterial lectin, called RegIIIγ, maintains mutualism between the gut microbiota and the host intestine. This material forms a thin layer as a physical barrier between the microbiota and the intestinal epithelial lining. However, losing this zone was associated with an increase in colonic bacteria and, consequently, activation of the immune response^40^. The paper by Sartor^31^, published in *Gastroenterology*, was the fifth most cited article. This review article highlighted a key topic of rationale for manipulating gut microbiota in the treatment of inflammatory bowel disease (IBD). Although this therapeutic strategy is uncontested, clinical studies do not follow standard criteria based on rigorous evidence for the use of these therapeutic modalities. However, some experts have proposed the use of ciprofloxacin and/or metronidazole or certain types of probiotics to treat IBD^31^.

## Future perspectives

The study of the links between the microbiota and antibiotic use is a rapidly evolving field and is expected to continue to be an area of intense research in the coming years. Based on the current trajectory of research related to the impact of antibiotics on the human microbiome and its consequences on health, there are several future perspectives to consider:

1. The study highlights that research on altered intestinal microbiota composition with antibiotic treatment was introduced after 2017. Therefore, it is necessary to explore the implications of such alterations in the gut microbiota for host health and how these changes can be mitigated.
2. The study also shows that research on gut microbiota and antimicrobial resistance has been ongoing for several years. However, further research is needed to investigate the mechanisms by which the microbiota contribute to antimicrobial resistance and how this knowledge can be utilized to develop new strategies to combat antibiotic resistance.
3. The study highlights the importance of probiotics as an alternative antimicrobial therapy, and it is necessary to further explore their potential to reduce antibiotic use and mitigate the negative effects of antibiotics on the gut microbiota.
4. The study emphasizes that various factors, including diet and stress, can disrupt the gut microbiota. Therefore, it is necessary to explore how these factors influence the composition of the gut microbiota and antibiotic efficacy and how this knowledge can be used to optimize antibiotic therapy.

## Strengths and limitations

This study is the first comprehensive bibliometric investigation of the links between antibiotics and microbiota research conducted worldwide. The study of the connection between antibiotics and the microbiota is still in its infancy, but it is expected to develop further soon. Therefore, scientific researchers, clinicians, and medical educators use the aforementioned development hotspots as a foundation and guide for developing new projects in their respective fields.

The current study has some limitations. First, we only extracted data from the Scopus database where it was necessary to do so to conform to the data formatting requirements of visualization tools such as VOSviewer. Nevertheless, the Scopus database, one of the most generally accessible and well-known resources globally, has been utilized in several previously conducted bibliometric research projects of a particularly high standard. Second, however, we are concerned with ‘antibiotics’ or ‘antimicrobials’, ‘antibiotics’ or ‘antimicrobials’, were those related to antibiotics per se rather than other related terminology, such as specific name or class of antibiotics. It is important to note that we must acknowledge the potential for bias in our sample selection due to the exclusion of certain publications that use specific antibiotic names in their titles. We are committed to ensuring that our research is as representative and unbiased as possible, and we urge caution in drawing conclusions from studies that may not have taken this into account. Third, the bibliometric analysis cannot directly assess the quality of the evidence presented in the publications. It can only provide a quantitative assessment of the impact of the publication, such as how many times it has been cited or the number of publications in a particular field. The quality of evidence presented in a publication depends on many factors, including study design, the methods used, and the validity of the results. Bibliometric analysis cannot assess these factors. Finally, the scope of the present study was limited to the title search and only contained search terms related to the microbiota and antibiotics. Therefore, it is possible that this study missed some articles that used the terms ‘microbiota’ and “antibiotics” or closely related terms as keywords or appeared anywhere in the text of the publication. However, if these false negative outcomes occur, their impact on overall results will be minimal^58,59^.

## Conclusions

This study is the first bibliometric analysis that objectively and thoroughly examines global microbiota trends related to antibiotic research over the past 20 years. In conclusion, this study provides a comprehensive summary of the current trends in global publications and helps researchers determine the current hot topics in this area. Currently, the number of publications in this field is increasing every year, with the United States and China making the greatest contributions. Furthermore, the *National Natural Science Foundation of China* and the *National Institutes of Health* provide significant support for this type of research. Therefore, it is recommended that researchers pay more attention to the latest promising hotspots, including altered intestinal microbiota composition with antibiotic treatment. Further research in these areas could provide new insights and strategies to maintain host health and combat antibiotic resistance.

## Data Availability

The data sets generated and/or analyzed during the current study are available upon request from the corresponding author.

## Abbreviations

GI: Gastrointestinal tract
IBD: Inflammatory bowel disease
